# Superoxide-producing lipoprotein fraction from Stevia leaves: definition of specific activity

**DOI:** 10.1186/s12906-019-2500-1

**Published:** 2019-04-25

**Authors:** A. S. Isoyan, K. V. Simonyan, R. M. Simonyan, M. A. Babayan, G. M. Simonyan, V. A. Chavushyan, M. A. Simonyan

**Affiliations:** 1 H. Buniatian Institute of Biochemistry NAS RA, 5/1 P.Sevag str, 0014 Yerevan, Armenia; 2 Orbeli Institute of Physiology NAS RA, 22 Orbeli Bros Street, 0028 Yerevan, Armenia

**Keywords:** Stevia leaves, NADPH-containing superoxide-producing lipoprotein (suprol)

## Abstract

**Background:**

*Stevia rebaudiana* Bertoni has various pharmacological actions, which includes antidiabetic, antioxidant, anti-inflammatory activities. The superoxide and consequently NADPH oxidase (Nox) are relevant targets involved in biological effects of Stevia. The presence of NADPH-containing superoxide-producing lipoprotein (suprol) in Stevia leaves has not yet been tested. The mechanism of producing superoxide radicals (O_2_^−^) by suprol was determined in vitro, which is associated with the electron transfer from NADPH in the composition of suprol by traces of transition metal ions (Fe^3+^ or Cu^2+^) to molecular oxygen, turning it into O_2_^−^. It is expected that the therapeutic efficacy of Stevia leaves is caused by specific activity of superoxide-producing lipoprotein fraction.

**Methods:**

For the first time, from the dry leaves of Stevia the NADPH-containing superoxide-producing lipoprotein was isolated and purified. The specific content of suprol (milligrams in 1 g of Stevia leaves- mg/g) was determined after desalination of suprol and lyophilization.

**Results:**

According to the method provided, the specific content of the isolated suprol from Stevia’s leaves was up to 4.5 ± 0.2 mg / g (yields up to 68.5 ± 4.7%, *p* < 0.05, *n* = 6). Nox forms a stable complex with suprol. The optical absorption spectrum of the Nox-suprol complex represents the overlapping suprol and Nox spectra, with a certain background increase and characteristic features of optical absorption for Nox. Due to O_2_^−^ producing capacity suprol-Nox complex discolors KMnO_4_ solutions, Coomassie brilliant blue, restores nitrotetrazolium blue to formazan and oxidizes epinephrine to adrenochrome. The oxidation activity of adrenaline is 50.3 ± 5.1 U / mg / ml (*p* < 0.05, *n* = 6).

**Conclusion:**

Superoxide-producing lipoprotein fraction-Nox complex from Stevia leaves (membranes) can modulate redox regulated signaling pathways and may play a positive role in type-2 diabetes by means of adrenaline oxidation mechanism.

## Background

*Stevia rebaudiana* Bertoni is a plant species in the genus Stevia of the sunflower family (Asteraceae), commonly known as candy leaf, sweet leaf, or sugar leaf. It has annual, subligneous, more or less pubescent stems with extensive, fibrous and filiform root system [1]. There are more than 100 species of stevia plant belonging to family Asteraceae, but *Stevia rebaudiana* Bertoni stands out for its excellent properties as a sweetener [2]. NADPH-containing superoxide-producing high-density lipoprotein fraction (suprol) is separated from the serum of mammals (human, rat, rabbit). The mechanism of producing superoxide radicals (O_2_^−^) by suprol was determined in vitro, which is associated with the electron transfer from NADPH in the composition of suprol by traces of transition metal ions (Fe^3+^ or Cu^2+^) to molecular oxygen, turning it into O_2_^−^ [3]. Native, inactivated suprol (in the absence of Fe^3+^ or Cu^2+^ ions) by means of electrons of NADPH groups exerts only reducing properties (does not produce O_2_^−^). Due to O_2_^−^ producing capacity suprol has anti-cancer efficacy in experimental models [4–6]. Plant NADPH oxidases, also known as respiratory burst oxidase homologues, are the most thoroughly studied enzymatic ROS-generating systems, which may serve as important molecular ‘hubs’ during ROS-mediated signalling in plants [7]. *Stevia rebaudiana* Bertoni, a natural sweetener plant having medicinal and commercial importance is used all over the world, as it manifests a wide range of activity, in particular antidiabetic, antioxidant, anti-inflammatory activities [8]. Steviol glycosides from the leaves of Stevia not only reduce the expression of genes of gluconeogenesis in the liver [9], increase the secretion and utilization of insulin, the sensitivity of cells to the effect of insulin on the experimental mouse model of insulin resistance caused by fructose [10], but also reduce the level of oxidative stress of liver [11]. Previous studies also found that Stevia’s utility is due to its antioxidant properties and the finding is supported by analysis of the phenols. Stevia contains a high percentage of phenols (up to 91 mg/g) and these constituents extracted from the leaves have been shown to display potent hypoglycemic activity [11]. Several studies demonstrate that polyphenols exert their biological activities by interaction with proteins and lipids. Rather, polyphenols may affect cell oxidant production and cell signaling and promote health. Natural polyphenols can modulate redox sensitive signaling by defining the extent of oxidant levels which can result in modification of cell signaling, function, regulating enzymes that generate superoxide, hydrogen peroxide and nitric oxide and regulating the activation of transcription factors sensible to oxidants [12]. Research has shown that plant phytosterols, structurally similar to cholesterol, compete with cholesterol for absorption and thus play a meaningful role in supporting healthy cholesterol levels [13]. It was shown that *Stevia rebaudiana* Bertoni contains the following sterols: stigmasterol (45.8%), beta-sitosterol (39.4%) and campesterol (13.1%) [14]. In our earlier studies in experimental model of type II diabetes caused by intensive consumption of dietary fructose we have shown that Stevia exhibits a membrane-stabilizing role of reducing increased level of total fractions of NADPH oxidase isoforms from central nervous system tissues and regulates NADPH-dependent O_2_^−^-producing activity [15]. The superoxide and consequently NADPH oxidase are relevant targets involved in Stevia biological effects. The presence of NADPH-containing superoxide-producing lipoprotein in Stevia leaves has not yet been tested. It is expected that efficacy of Stevia leaves is conditioned by specific activity of superoxide-producing lipoprotein fraction.

The aim of the work was to isolate the NADPH-containing superoxide-producing high-density lipoprotein fraction from Stevia leaves and determine its specific activity.

## Methods

Ecologically pure, high-quality raw material of Stevia (with a high content of biologically active substances), grown in Nagorno-Karabakh was harvested from August to October 2016. Stevia used in this study was botanically authenticated and voucher specimens (2779 A) were deposited in the Herbarium of Institute of Hydroponics (Experimental Hydroponic Station, outdoor hydroponic station with 60–100 m^2^ vegetation surface area (feeding scaffolds with 12–20 repetitions). All indices of safety of Stevia dry leaves and Stevia-based food are defined in Republican Veterinary and Phytosanitary Laboratory Services Center State non-commercial Organization (SNCO) (Ministry of Agriculture of the Republic of Armenia) (Yerevan, Erebuni 12, reg. N2780, 20.01.2014) and fully comply with the decision of the RA Government on “The approval of the technical regulation of the requirements for juices and juice products and the repeal of the decision N744 of the RA Government of 26 June 2009”.

Isolation and purification of suprol fraction was carried out using a previously developed method [3] with some modifications. In particular, after suprol (10 g) homogenization in water (100 ml) and centrifugation (for 10 min at 6000 g), the fraction of native suprol was precipitated adding 0.05 M HCI to pH 6.2. Further, the suprol precipitate was dissolved in 0.005 M potassium phosphate buffer (PPB) at pH 7.4 and after centrifugation was subjected to ion-exchange chromatography on a column with cellulose DE-52 (4 × 10 cm), balanced with 0.005 M PPB under the given conditions. Suprol does not precipitate on a column of DE-52 under these conditions, but was purified from traces of other accompanying proteins of acidic nature. Similarly, for the removal of the accompanying protein impurities of basic nature, suprol solution was subjected to ion-exchange chromatography on a glass column with cellulose KM-52 (suprol was not absorbed on a column). Thus, suprol fraction was purified from traces of protein impurities of basic nature. After concentration of suprol fractions by lyophilization, it was subjected to gel filtration on a column with Sephadex G-100 and balanced by 0.005 M PPB at pH 7.4. First fraction of native suprol was collected. This fraction was further passed through the Biogel P-150 at pH 9.5 and fraction with a symmetric elution diagram was collected (the solubility of suprol in water increases significantly at pH 9.5). After these purification procedures of suprol, value of the optical spectral index (А280/А430) was no longer increased and amounted to 8.1 ± 0.12 (*p* < 0.05, *n* = 6).

Removal of traces of Nox from suprol-Nox complex was carried out as follows. Suprol-Nox complex was precipitated from opalescent solution at pH 4.8 by centrifugation. Traces of KOH were removed and the sediment was washed twice with water (1:50). Hydrogen peroxide at a concentration of 0.05 M was added to a water solution of precipitate and incubated for 10 min at room temperature. Next, the precipitate was thoroughly washed with water to remove traces of hydrogen peroxide, which was determined by permanganometric titration. The sediment was homogenized in water at pH 9.5 and was subjected to ion-exchange chromatography on a column with cellulose DE-52 at рН 9.5.

**Gel electrophoresis** of suprol was performed in 15% polyacrylamide gel at pH 8.9 and 4.5 according to acidic and basic nature of the proteins associated with suprol. However, suprol under the influence of an electric current is aggregated prior to polyacrylamide gel electrophoresis. In the course of electrophoresis and protein staining, the presence of other water-soluble and suprol-associated proteins of acidic and basic nature was not observed on these gels.

**The specific content of suprol** (milligrams in 1 g of Stevia leaves-mg/g) was determined after desalination of suprol and lyophilization.

**The process of producing O**_**2**_^**−**^ **of suprol** was revealed by 4 different and well-known methods: the reduction of potassium permanganate, nitrotetrazolium blue, Coomassie brilliant blue and the oxidation of adrenaline to adrenochrome (E480 = 750 M^− 1^ cm^− 1^) [16–18]. The amount of suprol was considered per unit of O_2_^−^ -producing activity of suprol capable of enhancing the formation of adrenochrome to 50%. Specific O_2_^−^ -producing activity of suprol was expressed in u/mg suprol.

**The lipid composition** of suprol (8 mg) from suprol was determined by thin-layer chromatography. However, suprol is poorly soluble in organic solvents, which creates certain difficulties in the application of thin-layer chromatography [19].

**Ascorbate-dependent malonic dialdehyde** (MDA, a product of lipid peroxidation) was determined according to the method of Y. A. Vladimirov and others [20] determining the specific content of MDA (mg) in 1 mg of suprol. In particular, 1 ml of suprol (8 mg) was mixed with 1 ml of thiobarbituric acid (0.75%) and ascorbic acid (0.8 mM), heated at 50–60 °C for 30 min, then trichloroacetic acid (30%) was added and incubated at 90 °C for 30 min. After freezing and centrifugation, the amount of MDA in the supernatant was determined by measuring the density of the characteristic optical absorption of MDA at 535 nm (molar extinction of MDA at 535 nm is 1.56 × 10^5^ M^− 1^ cm^− 1^).

Statistical significance of differences in the results was assessed by student’s t-test (М ± m) with determination of criteria of reliability of obtained data. Significant differences between obtained values were analyzed using GraphPad Prism5.0 software (GraphPad Software, Inc., San Diego, CA).

Optical absorption and fluorescence spectra were recorded on a Hitachi-2000 (Japan) and Perkin Elmer (USA) spectrophotometer, respectively, at 20 in cuvettes with an optical path of 1 cm.

In the course of the research, K-70 and K-24 centrifuges (Janetzki, Germany) were used.

## Results

As shown in the methods, cell membranes of dry Stevia leaves were precipitated completely at рН 4.8. Optimal conditions for cleavage of hydrophobic bonds responsible for the retention of cell membrane components of Stevia occurred at рН 9.5, including incubation for 1 h at 37 ^o^ C. These components dissolved (a small amount) in water at рН 9.5 and formed an opalescent solution. The opalescent solution (contains components) was purified by gel filtration chromatography and ion-exchange chromatography at рН 9.5 as shown in the methods. There is a direct correlation between the process of aggregation of opalescent components and medium pH decrease. These indices are unique to lipoproteins**,** localized on the cell membranes, in particular Stevia cell membranes. Similarly, determination of lipid component, with the detection of malondialdehyde indicates that an opalescent solution (contains membranes of Stevia) is a NADPH-containing lipoprotein, as evidenced by results of various methods. Suprol (isolated from Stevia membranes) is associated with NADPH oxidase (forms a complex with Nox). This is evidenced by the results of gel filtration and ion-exchange chromatography. This complex is a natural formation of stevia cell membranes and verstile (is not broken down) by various cleaning methods.

**Fig. 1:**
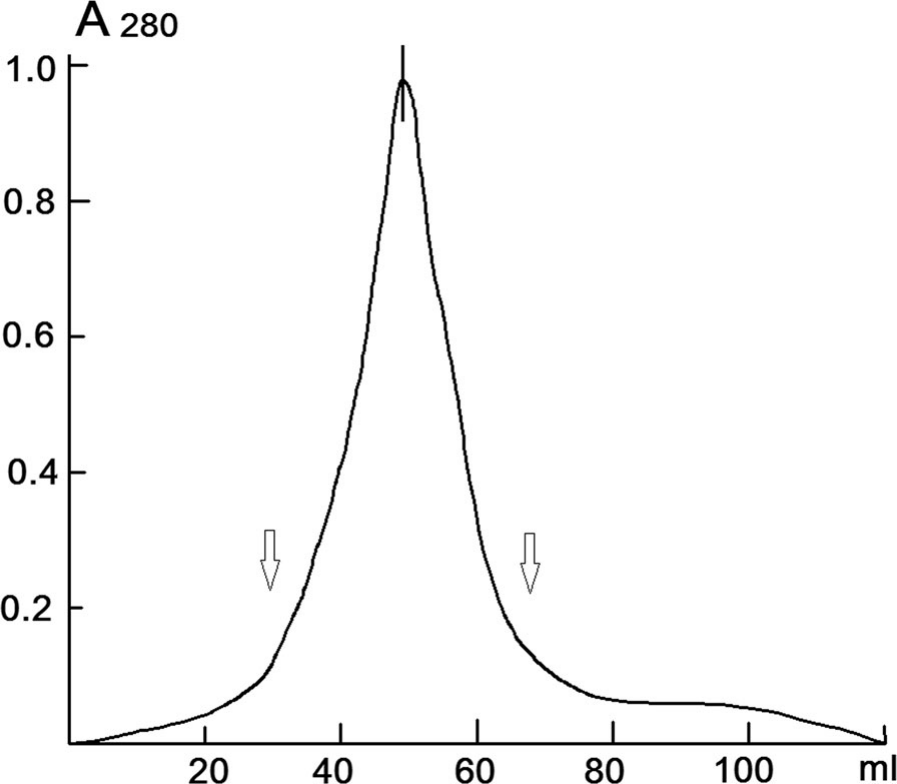
Chromatographic elution graph of suprol after loading on a column with Biogel P-150 at pH 9.5. For further research, the central fraction was used (indicated by arrows) (*p* < 0.05, *n* = 6)

According to the procedure described above, 10 g of dry Stevia leaves were isolated and purified to 45.3 ± 2.4 mg of suprol, with a specific content of Stevia leaves to 4.5 mg/g of and with an output of up to 68.5 ± 4.7% (*n* = 6, *p* < 0.05). The eluted peak has a symmetrical profile (Fig. 1). Chromatographic elution suprol graph after ion-exchange chromatography on columns with cellulose DE-52 and KM-52 and gel filtration on sephagex G-100 after gel filtration on Biogel P-150 at pH 9.5 (0.1 M KOH is added) has a symmetrical form (Fig. 1).

**Fig. 2:**
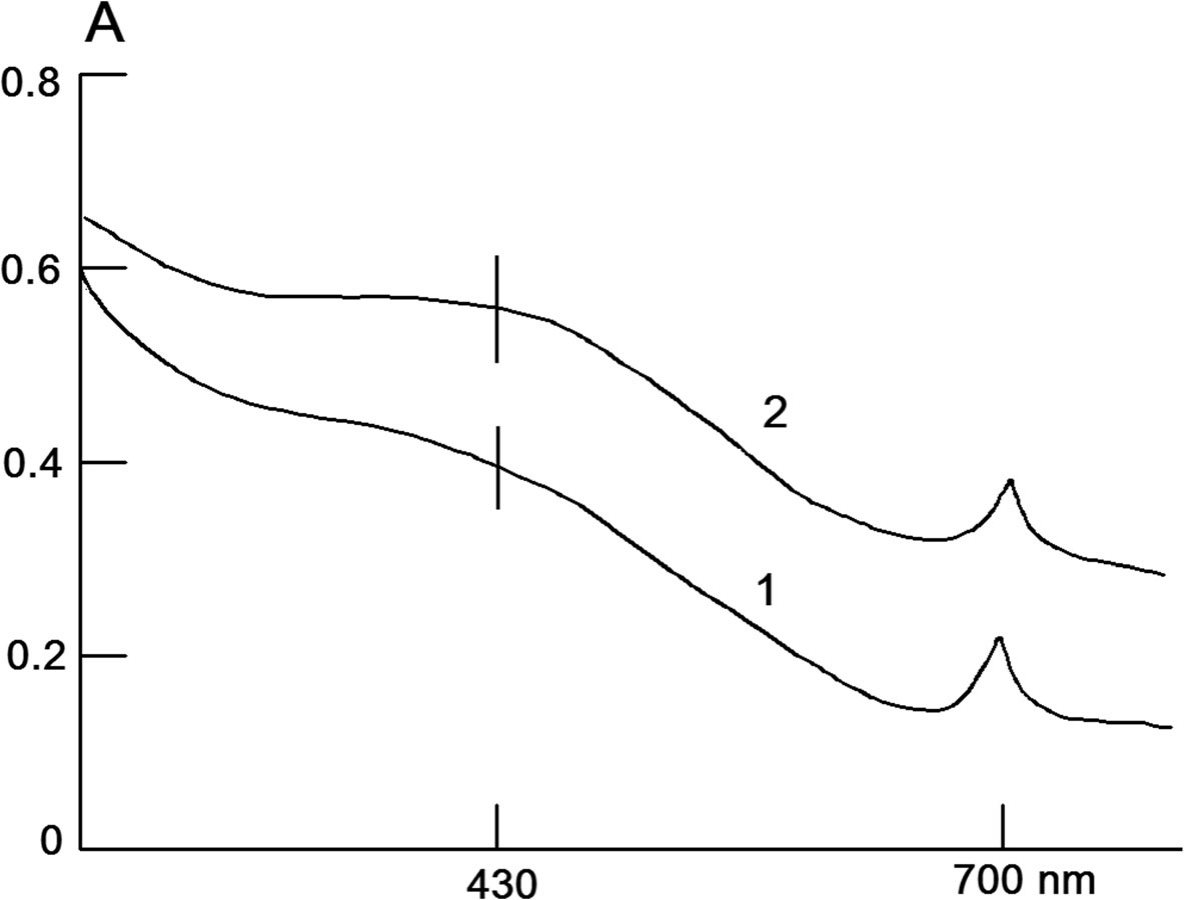
Optical absorption spectra of suprol dissolved in 0.005 M PPB at pH 9.5 within 2 h (1) and 30 h (2) at 4 °C after obtaining (*p* < 0.05 *n* = 6)

These data, as well as the results of electrophoresis indicate that the method described above results in generation of practically purified suprol associated fraction of Nox. After removal of traces of Nox from the suprol associated fraction by method described above, the degree of opalescence increases slightly, as Nox is a water-soluble protein and by means of association with suprol the solubility of suprol increases under the given conditions. The opalescence (opacity) of suprol becomes more pronounced during storage, due to self-aggregation, as shown in Fig. 2.

**Fig. 3:**
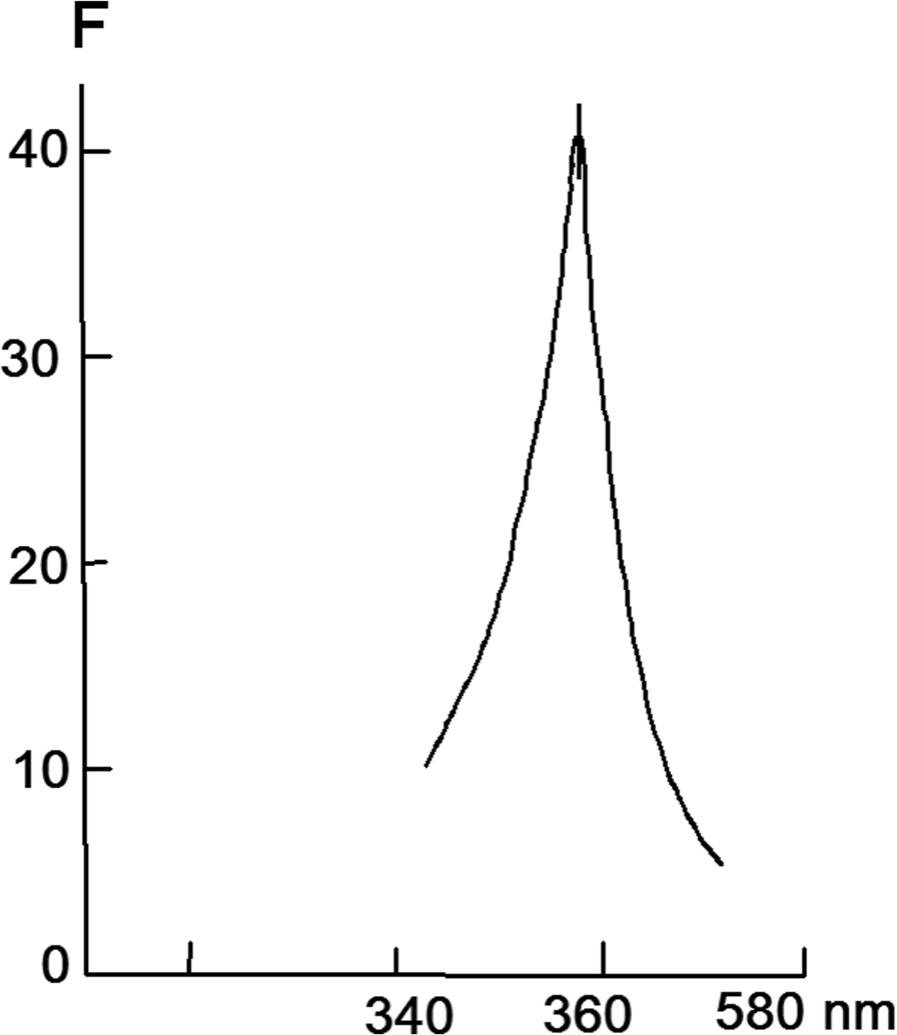
Optical absorption spectrum of Nox isoforms isolated from erythrocyte (1) and Stevia (2) membranes. Nox isolated from membranes of 10 ml of erythrocytes with a volume of 12 ml and from membranes of 10 g of dry Stevia leaves with a volume of 2 ml. Nox isoforms are dissolved in 0.1 M PPB, pH 7.4 (*p* < 0.05, *n* = 6)

After 28–30 h at 4 °C MDA content (14.3 μM / mg) increased 1.3–1.5 times. Lipid peroxidation process is suppressed (88–95%) by Cu, Zn-superoxide dismutase (4.10^− 8^ M) isolated and purified from beef liver. Optical absorption spectrum of Nox isoforms isolated from Stevia and erythrocyte membranes is shown in Fig. 3. As shown in Fig. 3, the form of an optical spectrum of Nox isoforms from erythrocyte and Stevia membranes are almost identical and have a maximum optical absorption in the visible range in the oxidized state (there are also characteristic absorption of these Nox after reduction by sodium dithionite at 558 nm).

**Fig. 4:**
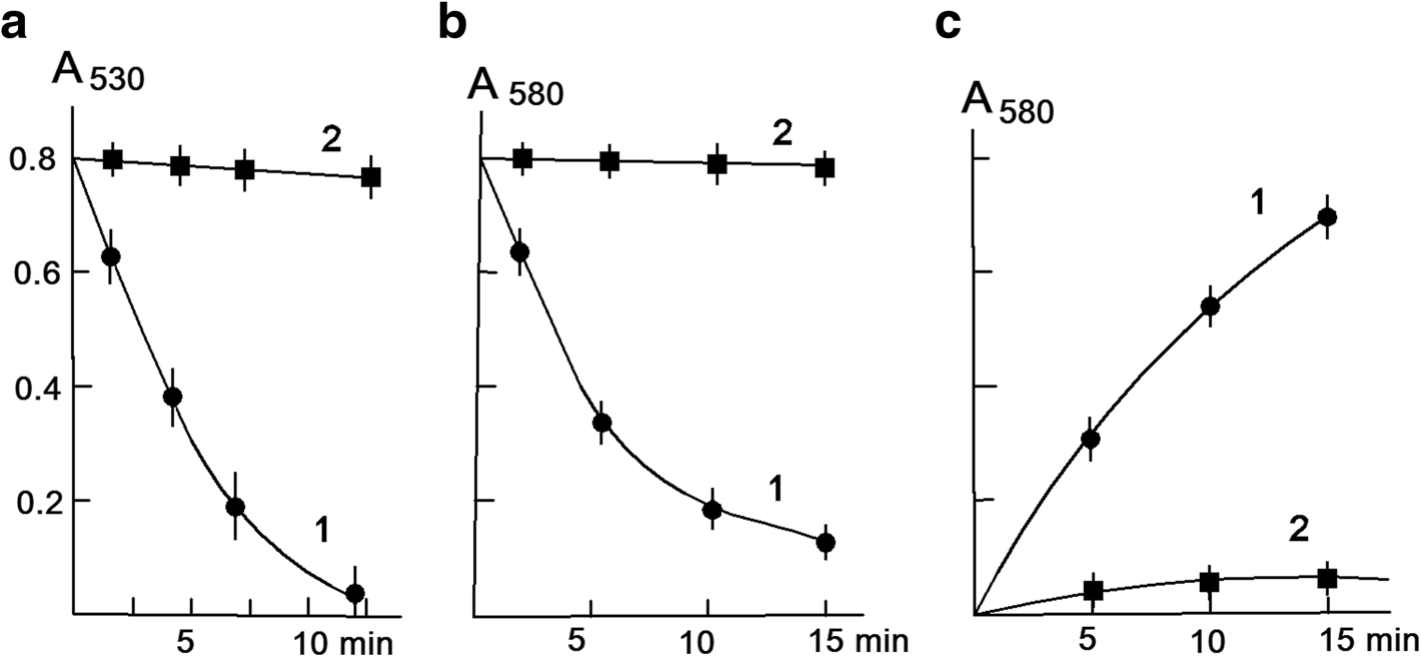
Kinetic curves for KMnO_4_ (**a**-1) and PPB (**b**-1) discoloration, reduction of nitrotetrazolium blue, with the formation of formazan (**c**-1) under the influence of 0.2 mg/ml suprol associated fraction of Nox in the absence of (1 -●-) and the presence of 5 × 10(− 8) M Cu, Zn-SOD (2 -■-), at a temperature of + 25 °C

As in the case of suprol from mammalian blood serum, there is significant protein absorption at 280 nm and a weak optical absorption at 430 nm, as well as a new sharp absorption peak at 690 nm (probably the latter absorption is characteristic of plant). The prevalence of sharp absorption peak is an important distinctive feature of plant suprol compared to serum suprol. However, the fluorescence spectrum of suprol has an emission peak of 430 nm with an excitation wavelength at 370 nm (Fig. 4) as in the case of suprol from mammalian blood serum. These indices are characteristics of NADPH fluorescence and indicate that suprol is a NАDPH containing high-density lipoprotein. Nox forms a stable complex with suprol. The optical absorption spectrum of the Nox-suprol complex represents the overlapping suprol and Nox spectra, with a certain background increase and characteristic features of optical absorption for Nox.

**Fig. 5:**
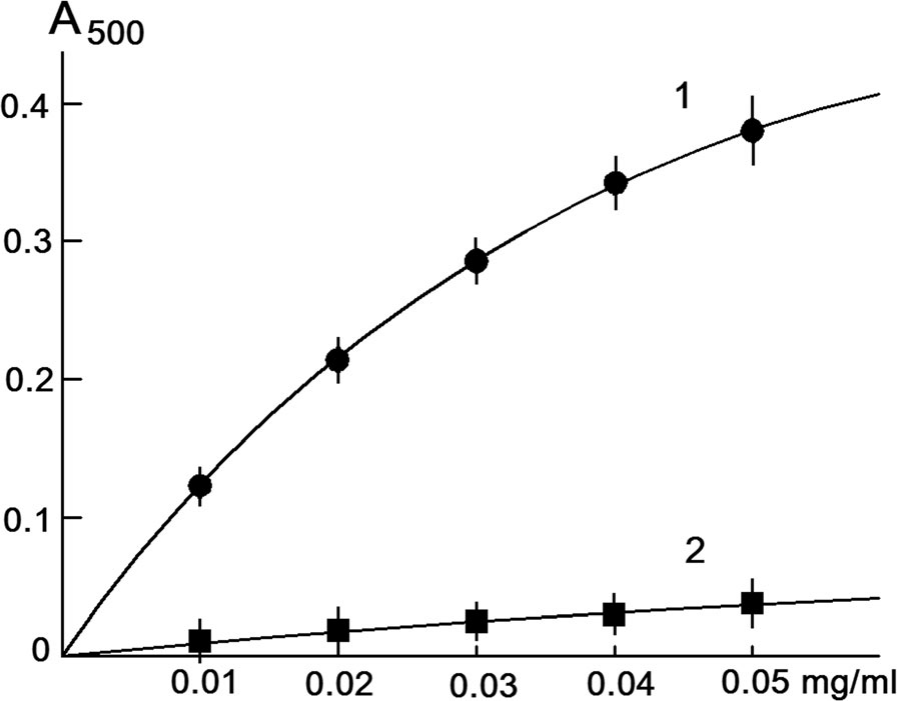
Formation of adrenochrome as a result of oxidation of adrenaline by superoxide radicals produced by suprol associated fraction of Nox of different concentrations at pH 7.4 in the absence of (1 -●-) and presence of 5.10^− 8^ M Cu, Zn-SOD (2 -■-)

Suprol-Nox complex produces O_2_^−^_._ Due to O_2_^−^ producing capacity suprol-Nox complex discolors KMnO_4_ solutions, Coomassie brilliant blue, restores nitrotetrazolium blue to formazan and oxidizes epinephrine to adrenochrome. These processes are suppressed by Cu, Zn-superoxide dismutase, as shown in Fig. 4. As shown in Fig. 5, native suprol (without activation by traces of exogenous Fe^+ 3^ or Cu^+ 2^ ions) oxidizes adrenaline (epinephrine). The rate of a reaction increases by 2.5–3 times, when the temperature of the reaction is raised to 35^о^С. Of particular interest is the effect of the oxidation of adrenaline (associated with suprol) at different concentrations of Nox (Fig. 5).

The estimated specific O_2_^−^-producing activity of adrenochrome formation (at 500 nm) as a result of adrenaline oxidation produced by O_2_^−^ is the suprol associated fraction of Nox (50.3 ± 5.1 u/mg/ml, *p* < 0.05, *n* = 6). Suprol nativity is based on the fact that organic solvents, detergents and ultrasound were not used in the isolation process, which in some way denaturates superoxide-producing proteins-enzymes [21, 22].

## Discussion

In lyophilized state Nox-suprol complex and suprol practically maintain their activity for 1–1.5 years. It is expected that administration of suprol in a body can stimulate the immune system by Nox association with membranes of immune cells [23]. On the other hand, a stable Nox –suprol complex from Stevia membranes as a natural and energetic source of O_2_^−^ can exhibit anti-cancer and bactericidal effects and can be used to determine new mechanisms of O_2_^−^ effects on different biosystems [24–26]. Thus, Nox-suprol complex is localized on the surface of the membranes of Stevia cells and suprol is an enzyme cofactor. In this case, the production of O_2_^−^ occurs directly, by electron transfer from NADPH to suprol-Nox complex, and then to O_2_, turning it into O_2_^−^. This is a new mechanism of O_2_^−^ production, unlike previously well-known cascade mechanism of an electron transfer from NADPH (cytosolic five isoforms of Nox) to terminal and active Nox isoforms located in the plasma membrane, then to O_2_, reducing to O_2_^−^ [27]. Oxygen-derived free radicals are important in both natural and acquired immunity [23]. It is expected that as a natural O_2_^−^ -producing system of suprol, stimulates both the immune system and shows a positive role, the mechanism of oxidation (cleavage) of adrenaline. Produced O_2_^−^ oxidizes adrenaline, which plays a pivotal role in glucose metabolism. Adrenaline causes an increase in level of glucose in the blood enhancing its formation in the liver [28]. It is expected that the oxidation of adrenaline by suprol is a possible mechanism by which contributes to the antihyperglycemic effect of Stevia leaves. It is noteworthy that polyphenols can modulate the activation of transcription factors sensible to oxidants. The latter includes the regulation of nuclear factor kappa-light-chain-enhancer of activated B cells (NF-kB) pathway. The regulation of the redox sensitive NF-kB pathway can be associated to the modulation of cell oxidant/antioxidant status. Besides being a redox sensitive transcription factor, NF-kB specifically regulates the expression of oxidant-generating enzymes, i.e. Nox1 and Nox4 [29]. This can generate a self-feeding cycle of NF-kB activation and increased oxidant production. A study on the insulin-resistant mice model shows that stevioside is also able to downregulate the NF-kB pathway, as well as enhancing whole-body insulin sensitivity, glucose infusion rate, and the level of the glucose-lowering effect of insulin [30].

Thus, suprol with the described distinctive features and related physico-chemical properties is closer to indices of suprol from the serum of mammals. Suprol nativity is based on the fact that organic solvents, detergents and ultrasound were not used in the procedure of its isolation, which in some way denaturates superoxide-producing proteins-enzymes [21, 22].

## Conclusion

In summary, we conclude that superoxide-producing lipoprotein fraction-Nox complex from Stevia leaves by specific activity apparently can regulate redox signaling pathways and may play a positive role in type-2 diabetes by means of adrenaline oxidation mechanism.

## Abbreviations

MDA: Malonic dialdehyde
NF-kB: nuclear factor kappa-light-chain-enhancer of activated B cells
Nox: NADPH oxidase
PPB: Potassium phosphate buffer
ROS: reactive oxygen species
suprol: Superoxide-producing high-density lipoprotein fraction
